# Paediatric Thoracic Imaging in Cystic Fibrosis in the Era of Cystic Fibrosis Transmembrane Conductance Regulator Modulation

**DOI:** 10.3390/children11020256

**Published:** 2024-02-16

**Authors:** Patrick W. O’Regan, Niamh E. Stevens, Niamh Logan, David J. Ryan, Michael M. Maher

**Affiliations:** 1 Department of Radiology, Cork University Hospital, T12 DC4A Cork, Ireland; 2 Department of Radiology, School of Medicine, University College Cork, T12 AK54 Cork, Ireland; 3 Department of Surgery, Mercy University Hospital, T12 WE28 Cork, Ireland; 4 Department of Medicine, Mercy University Hospital, T12 WE28 Cork, Ireland

**Keywords:** cystic fibrosis, radiography, computed tomography, magnetic resonance imaging, paediatrics, cystic fibrosis transmembrane conductance regulator modulator

## Abstract

Cystic fibrosis (CF) is one of the most common progressive life-shortening genetic conditions worldwide. Ground-breaking translational research has generated therapies that target the primary cystic fibrosis transmembrane conductance regulator (CFTR) defect, known as CFTR modulators. A crucial aspect of paediatric CF disease is the development and progression of irreversible respiratory disease in the absence of clinical symptoms. Accurate thoracic diagnostics have an important role to play in this regard. Chest radiographs are non-specific and insensitive in the context of subtle changes in early CF disease, with computed tomography (CT) providing increased sensitivity. Recent advancements in imaging hardware and software have allowed thoracic CTs to be acquired in paediatric patients at radiation doses approaching that of a chest radiograph. CFTR modulators slow the progression of CF, reduce the frequency of exacerbations and extend life expectancy. In conjunction with advances in CT imaging techniques, low-dose thorax CT will establish a central position in the routine care of children with CF. International guidelines regarding the choice of modality and timing of thoracic imaging in children with CF are lagging behind these rapid technological advances. The continued progress of personalised medicine in the form of CFTR modulators will promote the emergence of personalised radiological diagnostics.

## 1. Introduction

Cystic fibrosis (CF) is one of the most common progressive life-shortening autosomal recessive genetic conditions worldwide [1,2,3]. As a result of diagnostic and therapeutic advancements, life expectancy has greatly improved in recent decades [4]. The first description of cystic fibrosis as a pathological process was in the 1930s, and until 2012, CF management principally comprised the treatment of the clinical signs, symptoms and complications of the disease [5]. This involved various medical therapies, regular assessment by a comprehensive multidisciplinary team and regular diagnostic imaging for assessment of disease progression and acute exacerbations [6]. Ground-breaking translational research has generated novel therapies that target the primary cystic fibrosis transmembrane conductance regulator (CFTR) defect, known as CFTR modulators [7].

Mutations in chromosome 7 that encode the CFTR protein are the origin of the clinical manifestations of CF [8,9,10]. As a key component of normal physiological function, the CFTR protein is responsible for controlling the quantity and composition of epithelial secretions through its action as a cAMP-mediated chloride/bicarbonate channel [11]. A phenylalanine deletion at position 508 (F508del) is the most prevalent CF-causing mutation, being implicated in approximately 80% of CF cases worldwide [12]. Currently, there are >2000 CFTR genetic variants with a broad range of associated phenotypes and clinical severity.

CFTR mutations have been grouped into six categories based on the primary genetic defect: (I) no production of the protein, (II) abnormal protein folding and trafficking, (III) defective channel gating, (IV) decreased channel conductance, (V) decreased protein production and (VI) reduced stability at the plasma membrane [13]. Classes I–III generally result in minimal or no CFTR protein, with more severe disease. In classes IV–VI, there is usually some residual function and less severe disease. F508del, the most prevalent mutation, is a class II variant.

A mutated CFTR protein results in abnormal ion transport and dehydration of various epithelial surfaces, with subsequent accumulation of viscous mucus, chronic inflammation and remodelling of damaged tissue [14]. This pathological process is evident in multiple organ systems with varying degrees of severity and impact on day-to-day life, including respiratory and gastrointestinal symptoms, increased sweat chloride concentrations, infertility and exocrine pancreatic insufficiency [15]. Morbidity and mortality associated with CF are primarily mediated through chronic progressive lung disease [16]. CF patients are thought to have structurally normal lungs at birth. They then typically develop a neutrophilic inflammatory response secondary to recurrent airway infection with persistent mucous plugging, which progresses to develop into bronchiectasis and subsequently a decline in lung function. Early in the disease process, airway thickening, mucous plugging and air trapping may be present, while bronchiectasis may be mild [17,18].

CFTR modulators can restore the trafficking and folding of abnormal CFTR proteins (correctors) or enhance the channel opening probability (potentiators) when the protein is located on the plasma membrane [19]. Currently, four CFTR modulators are clinically approved by the European Medicines Agency (EMA) and the Food and Drug Administration (FDA) in the United States and the Medicines and Healthcare Products Regulatory Agency (MHRA) in the United Kingdom: ivacaftor, lumacaftor, tezacaftor and elexacaftor [20,21,22]. These medications have facilitated a paradigm shift in CF patient care.

The first disease-modifying medication approved in a paediatric population in CF was ivacaftor (tradename: Kalydeco^®^). Ivacaftor, which is licenced for use in patients as young as 1 month (FDA and MHRA) and 4 months (EMA), is indicated for use in a number of class III mutations and the R117H CFTR mutation, a class IV mutation [23]. It has been proven to promote increased lung function, decreased sweat chloride levels and improved nutrition in patients with at least one G551D mutation [24,25,26,27]. The prevalence of the G551D mutation in our Irish CF centre is as high as 23% [28]. Dual therapy in the form of lumacaftor–ivacaftor (tradename: Orkambi^®^) and tezacaftor–ivacaftor (tradename: Symkevi^®^) was then introduced [29,30]. Lumacaftor–ivacaftor, licenced for patients from 1 year, and tezacaftor–ivacaftor, licenced for patients from 6 years by the FDA, EMA and MHRA, are indicated for use in patients with a homozygous F508del mutation [31,32]. The addition of lumacaftor and tezacaftor demonstrated modest improvement in pulmonary function and sweat chloride concentration compared to ivacaftor treatment alone, thus supporting the concept of combination therapy [30]. This proof of concept led to large phase 3 clinical trials investigating the utility of triple combination therapy. Elexacaftor was added to the combination of tezacaftor/ivacaftor, resulting in an increased rate and magnitude of improvement in pulmonary function in the form of increased percent predicted forced expiratory volume in 1 s (ppFEV_1_), decreased sweat chloride concentration, increased subjective wellbeing, decreased pulmonary exacerbations and decreased hospital admissions [22,33,34]. This triple combination therapy is currently approved for patients that have at least one F508del mutation in the CFTR gene or a mutation in the CFTR gene that is responsive based on in vitro data by the FDA (tradename: Trikafta^®^) in patients 2 years and older [35]. The MHRA and EMA have approved its use (tradename: Kaftrio^®^) in patients with at least one F508del mutation. It is licenced for patients 2 years and older by the MHRA and 6 years and older by the EMA [36,37] (Table 1). As knowledge and safety data progress over time, the age inclusion for triple combination therapy is likely to decrease, as has been seen with ivacaftor.

In this article, we present a concise overview of thoracic imaging in paediatric patients with cystic fibrosis in the context of CFTR modulator therapy.

## 2. Methods

To provide a comprehensive overview of the topic, a narrative review was chosen as the study design. Medical literature searches were conducted using PubMed, Embase and Cochrane electronic databases. Search terms included paediatric cystic fibrosis, thoracic imaging and CFTR modulators, in addition to relevant MeSH terms and synonyms with the Boolean operators “and” and “or”. Inclusion criteria were broad and included English language human studies relating to CF and CFTR modulators. PWOR, NS and NL independently reviewed publication titles and available abstracts. Articles were assessed for study quality, methods and goals. Articles that were relevant to the aim of this review were included by consensus. No specific timeframe limit was applied in order to ensure a comprehensive search. Additional articles were included based on institutional knowledge. Themes were identified in the selected articles, and appropriate subsections were created.

## 3. Imaging

The landscape of CF and its associated diagnostics and therapeutics have developed significantly over time. Initially, early mortality was a result of abnormal pancreatic function, and few patients entered adulthood. Today, pancreatic dysfunction remains an important factor in CF management but is less likely to cause mortality directly [44]. 

After the development of appropriate therapeutics to effectively treat pancreatic dysfunction, it became clear that respiratory disease and its complications would be the largest contributors to CF-related morbidity and mortality going forward [45]. Centralising and standardising CF patient care and developing a consensus on management have led to improved patient outcomes [2]. A crucial aspect of paediatric CF disease is the development and progression of irreversible respiratory disease in the absence of overt clinical symptoms. Effective and accurate thoracic diagnostics have an important role to play in this regard.

### 3.1. Chest Radiography

Despite its limitations, many centres continue to utilise chest radiography in the primary assessment of the thorax in paediatric CF patients [2]. It has been established that even in adults with severe disease, chest radiographs provide limited value [46]. In children, chest radiographs are non-specific and insensitive in the context of subtle changes in early CF disease with computed tomography (CT), providing increased sensitivity [47]. Chest radiographs are readily available and low-cost, have been utilised by CF clinicians for decades and, as a result, still play a central role in many CF centres’ diagnostic armamentarium. 

It has been shown that even with the use of structured scoring systems, there is a high inter-observer variability inherent in chest radiographs [48,49]. In an effort to improve the diagnostic accuracy of chest radiographs, artificial intelligence has been utilised to calculate a Brasfield score on over 2000 paediatric chest radiographs with comparable accuracy to that of a paediatric radiologist [50]. However, implanted ports and peripherally inserted central catheters can confound these algorithms and pose a specific problem in the CF cohort [51].

Dynamic chest radiography (DCR) is a relatively novel, real-time cineradiographic imaging system utilising fluoroscopic images that allows for the identification and tracking of chest wall and diaphragm motion throughout the breathing cycle [52,53]. DCR has been utilised in adults after the commencement of CFTR modulator therapy and has identified significant increases in diaphragm range of motion, an increase in diaphragm speed and a decrease in projected lung area upon expiration, suggesting less air trapping. These findings support associated clinical improvements in pulmonary function tests following CTFR modulator therapy and potentially offer new relevant measurable metrics when assessing responses to CFTR modulation [54]. The utility of DCR in a paediatric population remains to be seen.

### 3.2. Computed Tomography

Structural lung changes are evident in CT at a very young age, with irreversible bronchiectasis identified despite stable pulmonary function tests (PFTs) or stable chest radiography [55,56,57]. PFTs represent a challenging task for young children to perform comprehensively, and they commonly underestimate the full extent of early CF disease [58]. Bronchiectasis has been identified on CT scans in infants as young as 3 months old and in up to 80% of CF children by the age of 5 years [59,60]. In a study looking at monitoring early lung disease in CF patients, there was a relationship between CT evidence of inflammatory airway disease in 5-year-olds and the development of non-reversable airway disease and bronchiectasis in adolescence. This suggests a time frame where intervention may prevent this progression. The presence of atelectasis on CT scans at 5 years was the strongest predictor of developing bronchiectasis over BMI, bronchiolar lavage or spirometry [61]. In the era of rapidly evolving CF therapies, now being licenced for younger age groups, there is even more need for the early diagnosis and monitoring of disease progression, where CT will continue to play a role [62]. CT imaging can facilitate the early escalation of treatment and is increasingly utilised in clinical trials as a reliable metric of response to intervention [63,64].

Up to one-third of children with CF will progress to satisfy the criteria for lung transplantation [65]. CT scores of CF severity are an independent risk factor for survival post lung transplantation [66]. There are multiple CT scoring systems for structural lung disease in CF, including Bhalla, PRAGMA and Brody [64,67,68]. Each of these scoring systems have their own advantages and limitations. Generally, the more comprehensive the scoring system is, the more time consuming and technically challenging it is to complete. These quantitative assessments of lung disease in CF have been shown to accurately correlate with improvements in lung disease in patients who have commenced CFTR modulation [69]. A French group has demonstrated in a cohort with a median age of 13.5 years (range 4–54 years) the feasibility of utilising artificial intelligence in quantifying the magnitude of disease burden in the lungs of CF patients on CT imaging [70]. The system was able to identify structural lung improvement in those patients undergoing CFTR modulator therapy and deterioration in those not in therapy. The benefit of this system is the quicker time frame to complete assessment, with the time to obtain AI quantifications in each lung exam being 2 min with good reproducibility, compared to a number of hours for a human conducting a similar assessment. Additional artificial intelligence has been developed to produce reader-independent quantitative outcomes in CT, such as airway tapering and the airway–artery ratio [71,72]. The inevitable emergence of commercially available software in paediatric thoracic imaging and the prospect of accurate CT scoring have the potential to revolutionise clinical practice [73].

Recent advancements in imaging hardware and software, including advances in iterative reconstruction, have allowed thoracic CTs to be acquired in paediatric patients at radiation doses approaching those of a chest radiograph [74,75]. The major disadvantage of low-dose CT imaging techniques is image noise and the resultant negative impact on image quality and the potential for “missed” imaging findings. Pure iterative reconstruction algorithms reduce image noise, improve the image quality of low-dose CT images, and allow up to an 80% dose reduction in CT [76]. Low-dose CT (LDCT) of the thorax and ultra-low-dose CT (ULDCT) of the thorax can now produce diagnostic-quality images with the capability for the detection and surveillance of imaging findings associated with lung disease [77,78]. Another practical method of reducing the radiation dose associated with CT of the thorax is to limit the number of phases of image acquisition. A large reduction in radiation dose can also be facilitated by only acquiring an end-expiratory CT as opposed to end-inspiratory and end-expiratory images [79]. When concern is generated regarding air trapping, the additional series can then be acquired [80]. Some authors advocate for the biennial screening of CF patients with LDCT of the thorax once the initial diagnosis has been established [81]. The potential future introduction of photon-counting CT has given rise to the hope that up to a 70% dose reduction is possible without significant image quality degradation [82,83].

Children with CF are more at risk with regard to ionising radiation given their inherent increased radiosensitivity, increased frequency of diagnostic imaging and now, in the era of CFTR modulators, increased life expectancy. The cumulative effective dose (CED) in CF patients has been steadily rising over several decades [6,84]. A reduction in radiation exposure without compromising clinical care or decision making is of paramount importance [85].

**Figure 1:**
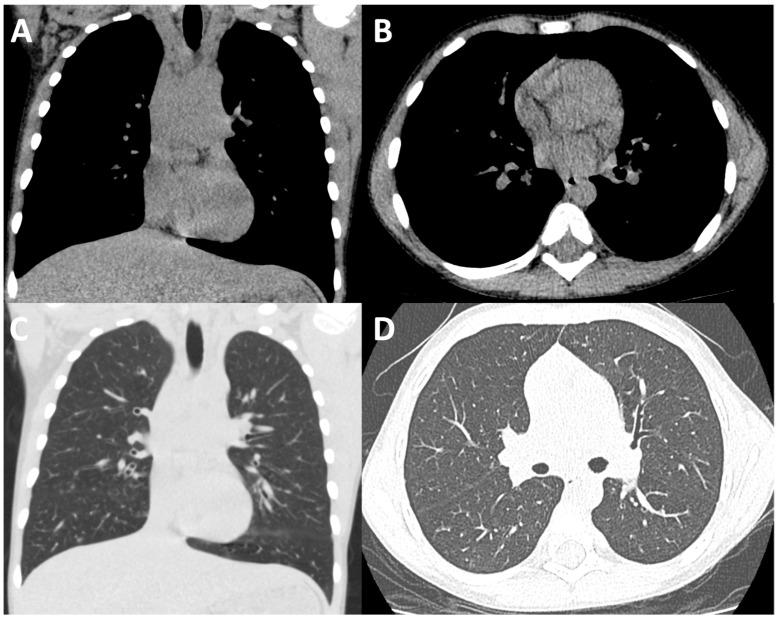
Conventional-dose CT of the thorax. Coronally (**A**,**C**) and axially reconstructed (**B**,**D**). Conventional-dose CT of the thorax images in soft tissue (**A**,**B**) and lung windows (**C**,**D**) in a paediatric male CF patient performed during an acute hospital admission. The dose length product (DLP) for this examination was 39.77 mGy*cm.

**Figure 2:**
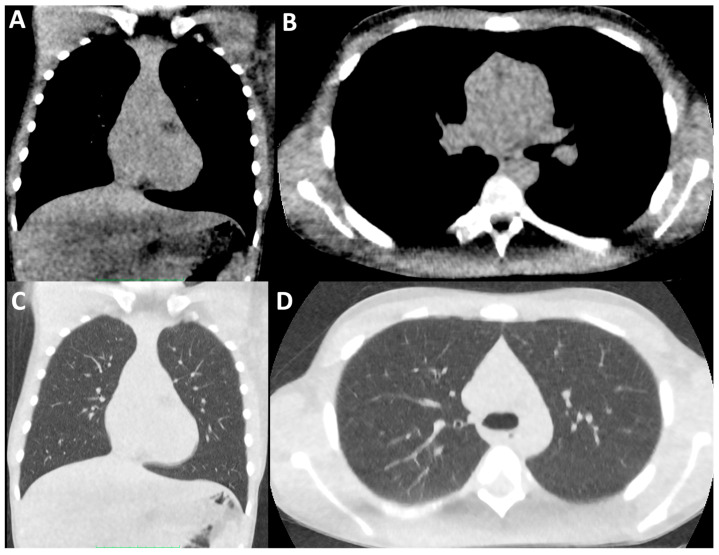
Ultra-low-dose CT of the thorax. Coronally (**A**,**C**) and axially (**B**,**D**) reconstructed ultra-low-dose CT thorax images in soft tissue (**A**,**B**) and lung windows (**C**,**D**) in the same paediatric male CF patient as Figure 1, performed during routine disease surveillance several years later. The DLP for this examination was 2.28 mGy*cm.

Despite these valid ionising radiation concerns, there is no internationally agreed ULDCT or LDCT thorax protocol for children with CF [86]. Our group has shown that the integration of ULDCT thorax protocols in favour of chest radiography can successfully be carried out without an increase in the cumulative effective dose in patients with CF undergoing CFTR modulation therapy [87]. Please see Figure 1 and Figure 2 demonstrating conventional-dose CT of the thorax and ultra-low-dose CT of the thorax, image quality and ionising radiation doses (39.77 mGy*cm vs. 2.28 mGy*cm, respectively) on the same male child with CF acquired several years apart.

### 3.3. Magnetic Resonance Imaging

Magnetic resonance imaging (MRI) of the thorax in children with CF can be performed to assess lung structure, provide functional lung information and demonstrate lung perfusion. Specific MRI techniques utilising ultra-short echo time acquisition have been developed to assess lung structure in people with CF [88,89]. Multiple research groups have demonstrated the comparability of MRI to CT in quantitatively assessing structural lung disease [90,91,92]. The limitations of MRI in the setting of paediatric CF include substantially longer study time vs. CT, the more threatening environment of MRI vs. CT with greater noise and a more enclosed and narrower tunnel. This can lead to respiratory motion artefacts and the need for sedation in paediatric patients. Other disadvantages include the limited standardisation of MRI acquisition techniques and more limited access to MRI infrastructure in comparison to CT.

Airway function can be assessed through MRI with the utilisation of inhaled or injected hyperpolarised material such as helium and, more recently, xenon-129. These hyperpolarised materials can increase the available signal by a magnitude of 10^5^ and allow the tracking of airway function during the acquisition [93,94]. These techniques carry the limitations of materials and infrastructure costs in addition to staff expertise [95]. Additional MRI techniques utilise the paramagnetic effect of oxygen in both room air (21%) and inhaled 100% oxygen to increase the signal-to-noise ratio [96,97,98]. These techniques require substantial post-processing ability, and standardisation in acquisition is needed before being widely utilised in a clinical setting [99].

Lung perfusion MRI to assess blood flow and subsequent gas exchange in chronically damaged lung tissue is also of clinical value [100,101]. Matrix-pencil decomposition MRI (MP-MRI) is feasible in young children as it only requires calm tidal breathing and not specific breath-holding manoeuvres, allowing for functional lung imaging of ventilation and perfusion without contrast, and has been used to reliably assess paediatric patients with CF [102]. Dynamic contrast-enhanced (DCE) MRI with the use of intravenous contrast (gadolinium) and arterial spin-labelled MRI without the use of intravenous contrast agents are available, with DCE MRI providing a better signal-to-noise ratio with the associated limitations of using gadolinium [103].

In the context of CFTR modulation with triple therapy, MRI has the ability to highlight improvements in pulmonary pathology that correlate well with clinical improvement in paediatric patients with CF [104,105].

In addition to thoracic imaging, MRI has utility in imaging CF-related liver, renal and cardiac disease [106,107,108]. Structural changes in the pancreas in pancreatic insufficient patients can be diagnosed and longitudinally followed with MRI [109].

### 3.4. Positron Emission Tomography (PET) CT

18F-Fluorodeoxyglucose (FDG) positron emission tomography (PET) CT is a well-recognised method of metabolic/functional imaging that can identify the inflammation and neutrophil burden associated with pulmonary exacerbations in CF [110]. PET CT can provide sensitive outcome metrics and has been successfully utilised in patients with CF to delineate chronic fibrosis from active infection [111]. However, the practical considerations of cost and availability, in addition to the additional substantial radiation exposure, have limited the use of PET CT in paediatric CF patient cohorts. 

In an attempt to avoid sedating young children to undergo the relatively time-consuming PET CT examination, ultra-short imaging protocols have been developed. Decreasing imaging time typically requires increasing the dose of radiopharmaceuticals to produce images of sufficient diagnostic quality, and the absence of sedation increases motion artefacts. The novel introduction of artificial intelligence-based reconstruction algorithms has facilitated a reduction in the radiopharmaceutical dose required to achieve satisfactory image quality in ultra-short PET CT imaging [112]. This recent technological advancement may allow PET CT to feature more regularly in the assessment of children with CF.

### 3.5. Ultrasound

Ultrasound is a safe, well-tolerated and cost-effective method of assessing the thorax in children without utilising ionising radiation [113]. A thoracic ultrasound in paediatric CF patients has the ability to identify pulmonary pathology such as consolidation and interstitial changes and correlates with spirometry. Ultrasound has a potential future role in disease surveillance [114]. The limitations of ultrasound include operator-dependency and the inability to image deep structures within the lung [115]. Further standardisation of imaging techniques and reporting guidelines will help progress the field of thoracic ultrasound. Despite the absence of ionising radiation and the widespread availability of ultrasound, there are currently insufficient data to support the routine inclusion of thoracic ultrasound in the diagnosis and surveillance of CF [116,117].

## 4. Discussion

With increasingly milder CF disease in paediatric and adult patients and the licencing of CFTR modulators for infants as young as 1 month who may have no clinical symptoms or abnormal lung function tests, there is a need for sensitive outcome measures that can detect early structural lung changes for both clinical trials and routine clinical follow-up. There is an increasing number of female CF patients seeking pregnancy, and fertility has been shown to improve with CFTR modulator therapy [118,119]. There has been evidence of placental and breastmilk transfer of CFTR modulators with potential subsequent decreased disease burden in infancy, which may also require further monitoring [120]. The early detection of pulmonary changes is important to facilitate timely and appropriate treatment with the ultimate aim of limiting and delaying disease progression, considering pulmonary disease is the primary cause of morbidity and mortality in CF patients [121,122]. While MRI has been shown to be a suitable tool for measuring responses to CFTR modulators and has the benefit of no exposure to radiation, the practical challenges and limited access, coupled with the low sensitivity of chest radiographs, means that CT will maintain its central position in CF thoracic imaging for the foreseeable future. There are ongoing attempts to create even more effective CFTR modulators, and whether these will change the outcomes for CF patients is yet to be seen [123]. 

CFTR modulators are within the novel field of personalised medicine and are only possible through the development of high-volume genetic sequencing technologies and subsequent targeted therapies [124]. In such a rapidly developing field, there are reasonable concerns regarding the ethical considerations of genetic data management, the procedures for managing incidental genetic findings and equitable access to the benefits of personalised medicine [125]. Multiple European studies have demonstrated heterogenous access to advanced biomarkers with the overarching goal of harmonising data-sharing systems, diagnostic frameworks and decision-making models [126,127]. Whether due to the absence of a suitable genetic defect or limited access to expensive CFTR modulation therapy, non-modulated CF patients can be expected to follow a clinical path with more severe disease. Traditional diagnostic and therapeutic regimes will remain relevant for this patient cohort.

Asymptomatic children are a particularly important population to monitor as there is the possibility of intervening before bronchiectasis develops [61]. Another consideration is that there have been cases in adults where doses of CFTR modulators have been reduced in response to side effects, particularly anxiety and neurocognitive effects. The evidence to date has shown lesser side effects in response to dose reduction with ongoing clinical benefit, measured with spirometry and sweat chloride levels [128,129,130]. It follows that there may be a role for imaging surveillance in children who may require CFTR modulator dose reduction and are unable to complete spirometry or in those with normal spirometry.

Pulmonary exacerbations have been shown to impact lung function in CF, with up to 50% of patients who exacerbate failing to return to their baseline pulmonary function [131,132]. CFTR modulators slow the progression of CF, reduce the frequency of acute exacerbations and significantly extend life expectancy. In this context, children with CF will undergo diagnostic imaging earlier. The mean age for a first CT scan has dropped from 20 years in patients born before 1980 to 1.9 years in patients born after 1997 [84]. As such, CF patients are also more likely to undergo surveillance imaging for a longer period of time than ever before in order to assess thoracic disease burden and adjust treatment accordingly. The major limitation to the use of CT for the surveillance of CF lung disease is the associated radiation exposure of conventional CT of the thorax vs. chest radiography (3.5 vs. 0.02 mSv) [133]. Care providers must remain aware of the carcinogenic risks of ionising radiation and the widely accepted linear no-threshold model that states that the more radiation delivered to patients, the greater the risk of resulting carcinogenesis [134]. The increasing availability of ULDCT will eliminate this major obstacle for CT utilisation, and the use of CT in CF will likely continue to sharply increase. It is important to keep radiation exposure to a minimum while not compromising the quality of diagnostics. Another common concern in paediatric radiology is the requirement for sedation or general anaesthesia and the associated risks. One study looking at LDCT without anaesthesia in paediatric patients (mean age: 66 months) to assess a variety of pulmonary diseases found that satisfactory diagnostic quality was achieved in 98.9%, of which 82.6% were considered excellent. Only one case had blurring, moderately compromising the image. Furthermore, 51.2% of this cohort went on to be diagnosed with CF [135]. More prospective studies to further assess surveillance with ULDCT or LDCT without anaesthesia in the CF paediatric population would be of value, but the evidence to date is encouraging. 

It is important to be cognisant of the fact that CF is a multi-system pathology, and throughout their lives, patients may require abdominal imaging that will result in an increased CED. Patient radiation dose tracking may play a role in the future care of CF patients. An initiative that automatically records all medical radiation exposures, which may then be included in patients’ radiology reports and health care records, has been developed. This highlights those patients at risk of a high CED and allows for corrective actions [85]. Radiation dose tracking software has been shown to significantly reduce the dose length product in paediatric populations and may allow for a comparison of performance between institutions and ease the adoption of best practices [136,137]. Another important factor to consider is the patient’s or parent’s understanding of radiation risk. It is known that there is increased media reporting on this topic, and there is a relative high volume of inaccurate information [138]. It has been suggested that there be a move to informed decision making, allowing decisions to be made in conjunction with patients or parents, keeping in mind the known and unknown factors of radiation risks [85]. 

Thoracic MRI has been shown to be accurate in monitoring the response to CFTR modulator therapy, and in the paediatric population in particular, it has the added benefit of no radiation exposure. One of its limitations is access to the various resources required. It has been suggested that with further prospective trials, patients that may benefit most from MRI surveillance, for example, those with mild disease, may be identified, and this could allow for an ideal balance between sensitivity, the allocation of resources and practicality [102]. The assessment of ventilation, perfusion and structure in a single examination leads to the hope that MRI will assume a greater role in the care of children with CF as availability increases and techniques are standardised.

The model of CF care based on a centralised “CF Centre” has improved morbidity and mortality for CF patients [19]. The multidisciplinary team is an essential element of CF patient care. This team includes CF-specialised physicians, clinical nurse specialists, physiotherapists, psychologists, pharmacists, researchers and radiologists with an ever-evolving role in patient care. It is vital that the clinical team maintain close links with associated specialities, ranging from clinical geneticists to assisted fertility services and transplant teams, to provide a comprehensive and up-to-date service [139]. Interdisciplinary collaboration is the foundation of best practice guidelines established by the European Cystic Fibrosis Society [140]. In a study assessing research priorities in a cohort including patients, their families and healthcare professionals, simplifying the treatment burden for CF patients was one of the top ten priorities [141]. While defining the most sensitive and effective surveillance methods and the timing of these investigations is considered important, it is also important to balance this with the impact of treatment and care on these patients and their families [142]. The MEASTRO group has recommended the establishment of international guidelines to outline the optimal modality and timing of radiological investigations for initial diagnosis, ongoing surveillance and acute exacerbations of CF [143].

This narrative review has several limitations. The discussed literature was limited to the English language and may not fully represent published data. There were relatively heterogenous sample sizes, research methods and research goals throughout the included studies, limiting the cohesiveness of these data. These data are specific to paediatric patients with CF and may not be generalisable to other patient cohorts. The utilisation of advanced imaging hardware and software is not universally available to all healthcare systems, which limits the general applicability of this review. 

When considering the future directions of this field, formal international guidelines regarding the choice of imaging modality and timing of thoracic imaging in children with CF are lagging behind the rapid technological advances in radiological hardware and software. LDCT, ULDCT and, where available, thoracic MRI, will assume more pivotal roles and will likely be recommended to be performed more frequently in place of chest radiography in future CF radiological diagnostics and associated guidelines.

The continued progress of personalised medicine in the form of CFTR modulators should prompt the development of personalised radiological diagnostics in the form of adjusted follow-up intervals to conform with disease severity and imaging protocols adjusted to body composition to optimise accuracy while minimising radiation dose.

## 5. Conclusions

In the era of CFTR modulation, in conjunction with advances in CT imaging techniques, LDCT and ULDCT of the thorax will establish an increasingly pivotal role in the routine care of children with CF. LDCT and, to a much lesser extent, ULDCT confer a decreasing penalty in radiation dose compared to chest radiography, with significant improvements in sensitivity and specificity in disease identification, allowing for earlier escalation in treatment and improved patient outcomes.

## Figures and Tables

**Table 1:** Summary of CFTR modulators.

CFTR Modulator	Mutations Targeted	EMA	FDA	MHRA
Ivacaftor (KALYDECO) (improves the activity of the defective CFTR protein)	Indicated for use in CF patients with R117H CFTR mutation or one of the following gating (class III) mutations in the CFTR gene: G551D, G1244E, G1349D, G178R, G551S, S1251N, S1255P, S549N or S549R [23,38,39].	First approval—2012 Currently approved for infants at least 4 months and > 5 kg [23].	First approval—2012 Currently approved for infants at least 1 month [39].	First approval—2012 Currently approved for infants at least one month, toddlers and children >3 kg [38].
Lumacaftor–ivacaftor (ORKAMBI) (lumacaftor increases the number of CFTR proteins on the cell surface)	Indicated in patients who are homozygous for the F508del mutation in the CFTR gene [31,40,41].	First approval—2015 Currently approved for patients 1 year and older [40].	First approval—2015 Currently approved for patients 1 year and older [31].	First approval—2015 Currently approved for patients 1 year and older [41].
Tezacaftor–ivacaftor (SYMKEVI) (tezacaftor increases the number of CFTR proteins on the cell surface)	Indicated in patients homozygous for the F508del mutation or who are heterozygous for the F508del mutation and have one of the following mutations in the (CFTR) gene: P67L, R117C, L206W, R352Q, A455E, D579G, 711 + 3A → G, S945L, S977F, R1070W, D1152H, 2789 + 5G → A, 3272-26A → G and 3849 + 10kbC → T [32,42,43].	First approval—2018 Currently approved for patients 6 years and older [42].	First approval—2018 Currently approved for patients 6 years and older [32].	First approval—2018 Currently approved for patients 6 years and older [43]
Elexacaftor–tezacaftor–ivacaftor (KAFTRIO/TRIKAFTA) (elexacaftor increases the number of CFTR proteins on the cell surface)	Indicated in patients who have at least one F508del mutation in the CFTR gene [35,36,37].	First approval—2020 Currently approved for patients 6 years and older [37].	First approval—2019 Currently approved for patients 2 years and older [35].	First approval—2020 Currently approved for patients aged 2 years and older [36].

CFTR (cystic fibrosis transmembrane conductance regulator); EMA (European Medicines Agency); FDA (Food and Drug Administration); MHRA (Medicines and Healthcare Products Regulatory Agency).

## Data Availability

No new data were created or analyzed in this study. Data sharing is not applicable to this article.
